# Detection of Triacetone Triperoxide (TATP) and Hexamethylene Triperoxide Diamine (HMTD) from the Gas Phase with Differential Ion Mobility Spectrometry (DMS)

**DOI:** 10.3390/s21134545

**Published:** 2021-07-02

**Authors:** Mirosław Maziejuk, Monika Szyposzyńska, Aleksandra Spławska, Monika Wiśnik-Sawka, Michał Ceremuga

**Affiliations:** 1 Military Institute of Chemistry and Radiometry, al. gen. A. Chruściela “Montera”, 105, 00-910 Warsaw, Poland; m.maziejuk@wichir.waw.pl (M.M.); a.splawska@wichir.waw.pl (A.S.); m.wisnik-sawka@wichir.waw.pl (M.W.-S.); 2 Military Institute of Armament Technology, ul. Prym. St. Wyszyńskiego, 7, 05-220 Zielonka, Poland; ceremugam@witu.mil.pl

**Keywords:** differential ion mobility spectrometry (DMS), ion mobility spectrometry (IMS), explosives, triacetone triperoxide (TATP), hexamethylene triperoxide diamine (HMTD)

## Abstract

One of the significant problems in the modern world is the detection of improvised explosives made of materials synthesized at home. Such compounds include triacetone triperoxide (TATP) and hexamethylene triperoxide diamine (HMTD). An attempt was made to construct an instrument allowing for the simultaneous detection of both compounds despite the large difference of vapor pressure: very high for TATP and very low for HMTD. The developed system uses differential ion mobility spectrometry (DMS) in combination with a specially designed gas sample injection system. The created system of detectors allowed for the detection of a high concentration of TATP and a very low concentration of HMTD. TATP detection was possible despite the presence of impurities—acetone remaining from the technological process and formed as a coproduct of diacetone diperoxide (DADP) synthesis. Ammonia added to the carrier gas improved the possibility of detecting the abovementioned explosives, reducing the intensity of the acetone signal. The obtained results were then compared with the detection capabilities of drift tube ion mobility spectrometer (DT-IMS), which has not made possible such detection as DMS.

## 1. Introduction

The development of the detection of explosives is important for military and security purposes due to the use of explosives in terrorist attacks. This is especially important for materials that can be manufactured at home. Such materials include triacetone triperoxide (TATP), which was used in the attack in July 2005 in London, and hexamethylene triperoxide diamine (HMTD), which was used in the terrorist attack in December 1999 in the USA. Explosives detection is based on two strategies: the detection of substance vapors and particle analysis after swipe sampling. Vapor detection is faster as no sampling or preparation is required. However, vapor analysis is more challenging due to the low amount of substance evaporated from the surface. The vapor pressure (VP) is the pressure of a gas above the surface of a substance. This value for explosives varies from a single hPa to 10^−15^ hPa.

Vapor pressure measurement is difficult due to slight signal variations. The determination of VP is performed at an elevated temperature, which may contribute to material decomposition or molecular desorption from the surface, which may result in the appearance of additional analytical signals. Vapor pressure can be determined by a dynamic method consisting of mass change measurements of a substance at a constant temperature [1,2] and static methods, such as manometric VP determination [3] or surface phase studies using gas chromatography [4,5].

**Figure 1:**
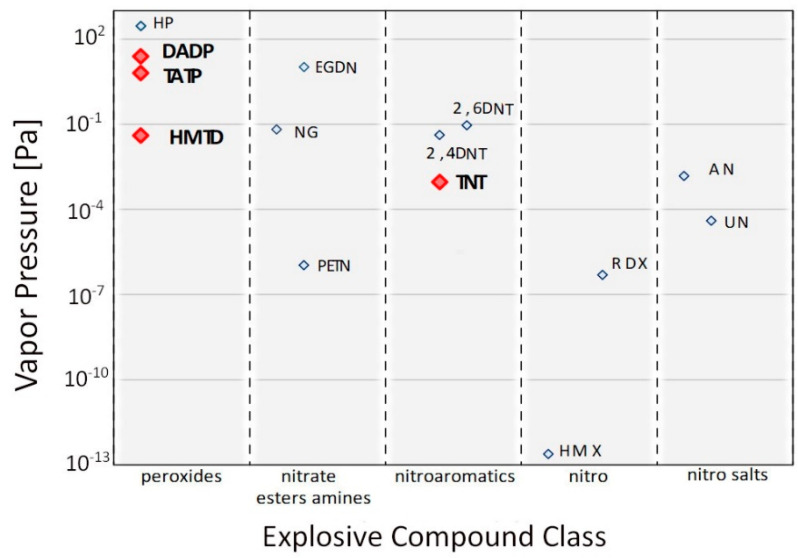
Vapor pressure values for various groups of explosive compounds (Reprinted from [12]).

Many studies have investigated the vapor pressure of TATP [2,6,7,8,9], diacetone diperoxide (DADP) [1,2] and HMTD [4,10,11]. Ewing and colleagues [12] compiled the values of the vapor pressure of explosive substances available in the literature and standardized them by determining their average values. Figure 1 presents the VPs for various explosives.

DADP is a peroxide with the highest VP, which is 24.7 Pa; for TATP, this value is 6.39 Pa. The vapor pressure of these substances is compared to that of liquid nitro derivatives such as nitroglycerin (NG). HMTD has a vapor pressure 150 times lower than that of TATP, which is 3.9 × 10^−2^ Pa. These values are significantly higher than those of commonly used strong grinding materials such as octogen (HMX) or hexogen (RDX). The vapor pressures of the described acetone peroxides are higher than that of TNT, 9.27 × 10^−4^ Pa.

Since TATP and HMTD do not contain nitro or aromatic functional groups, analytical devices for conventional explosives are unsuitable for detecting them. In recent years, many techniques of TATP and HMTD analysis have been developed, including infrared (IR) [13,14], liquid chromatography (LC) [15,16,17] and IMS detectors, which play a significant role in detecting these materials [18,19,20,21].

### 1.1. IMS Technique

**Figure 2:**
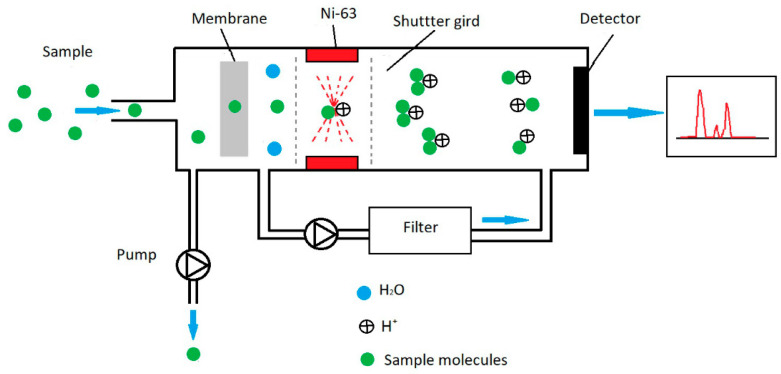
Operating principle for ion mobility spectrometry (IMS).

Ion mobility spectrometry (IMS) uses differences in ion mobility in carrier gas under the influence of an electric field [22,23,24,25,26] (Figure 2). A classical IMS (drift tube ion mobility spectrometer, DT-IMS) spectrometer has a weak linear electric field with values from 150 to 400 V/cm. Within these fields, ion mobility is constant.

Ion separation in a homogeneous electric field takes place based on differences in their mobility. The results obtained from the detector are presented in the form of drift time spectra. An IMS detector with a membrane was used in the laboratory research.

### 1.2. DMS Technique

A variant of IMS is differential ion mobility spectrometry (DMS) [27,28,29,30,31]. In DMS, ions are distinguished because of the difference between mobilities at high and low electric fields [32,33]. For strong electric fields (exceeding 12 kV/cm), ion mobility depends on the applied field, and the dependence is nonlinear. The relationship between ion mobility and the intensity of the electric field is expressed by Equation (1) [34].

(1) $$K(E/N)=K_{0}\cdot [1+\alpha (E/N)]$$

where *K*_0_ is the reduced ion mobility [cm^2^V^−1^s^−1^], *E*/*N* is the electric field in Townsend units (1 Td = 10^−17^ V·cm^2^), which is known as the normalized molecular density, and *α*(*E*/*N*) is the normalized function describing the electric field mobility dependence.

**Figure 3:**
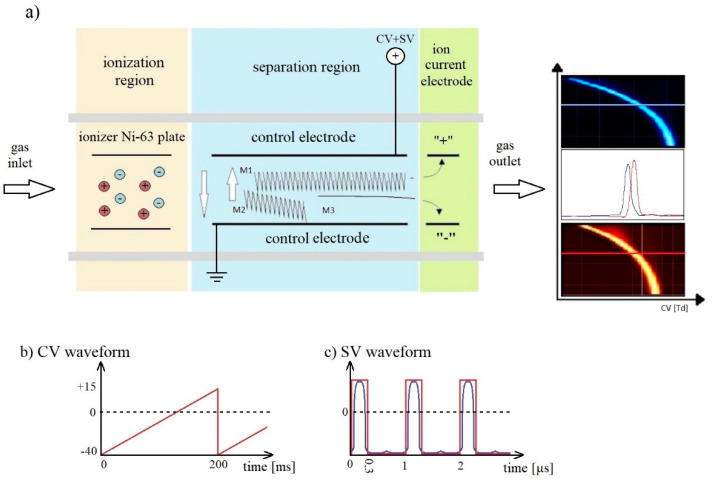
Principle of differential mobility spectrometry. (**a**) Schematic diagram of DMS spectrometers; (**b**) CV waveform; (**c**) SV waveform.

DMS spectrometers consist of an ionization area and a space in which ions are separated (Figure 3) [35,36]. An isotopic source emitting β particles is placed in the ionization region. Carrier gas transports generated ions through the separation region, which consists of two parallel plates on which metal electrodes are placed, the distance between electrodes (gap) should be almost 0.5 mm. The electric field produced by these electrodes is perpendicular to the gas flow direction. The voltage between the electrodes consists of a variable component called the separation voltage (SV) and compensation voltage (CV).

The waveform of SV is shown in Figure 3c: it should be a rectangular wave with 30% of duty; practically it is very similar to a dotted line. Voltage should be regulated from 400 V to 2000 V, 1 MHz or above should be applied; if the frequency is too low we cannot seen the light ions.

The compensation voltages are shown in Figure 3b as a very low wave. These voltages are generated as a special electric field which works as an ion filter.

In the established conditions only one type of ion comes through the control electrode to the ion current electrode. In the time t1 the mobility of ions M1 is K1 and in the time t2 it is K2, but the compensation voltage reduces these differences and allows ion M1 to reach the collecting electrode. The other ions going to the control electrode cannot be observed via the collecting electrode. Slowly changing the compensation voltage allowed us to make a graph (function ion current depending on compensation voltage) of all ions generated in the ionization region. This allowed us to order from higher to lower the values of α (differences of mobility).

The analyte introduced into the IMS/DMS detector is ionized by charge transfer (proton) between the reaction ions and the neutral molecule of the analyte. The change in the enthalpy of this reaction taking place in the gas phase is called proton affinity (PA). For the ionization of each substance, the values are different for TATP and HMTD. They are 860 kJ/mol [37] and 940 kJ/mol [11], respectively.

## 2. Materials and Methods

The DMS chamber used to build the measuring system was made of ceramic substrates [32]. The gas was ionized with an electrode made of nickel isotope (^63^Ni). The detector drift area consisted of two parallel electrode plates (5 × 25 mm) placed 0.635 mm apart. A high-amplitude 3 MHz asymmetric voltage waveform was generated by the HSV generator. Voltage was applied to the electrodes across the flow direction of the carrier gas. The applied voltage range of separation was from 7 Td to 118 Td (peak to peak). A constant weak compensation voltage ranging from −5.4 Td to +2.1 Td was applied to the electrodes. At the end of the drift section, two collecting electrodes (5 × 5 mm) were installed, which in the DMS detector allowed the simultaneous obtaining of a signal from positive and negative ions, as shown in Figure 3. Measurements were made using air as carrier gas, dried with 13 X molecular sieves (Merck, France). Internal gas flow through the detector was 3.2 L/min. The detector temperature was 45 °C.

**Figure 4:**
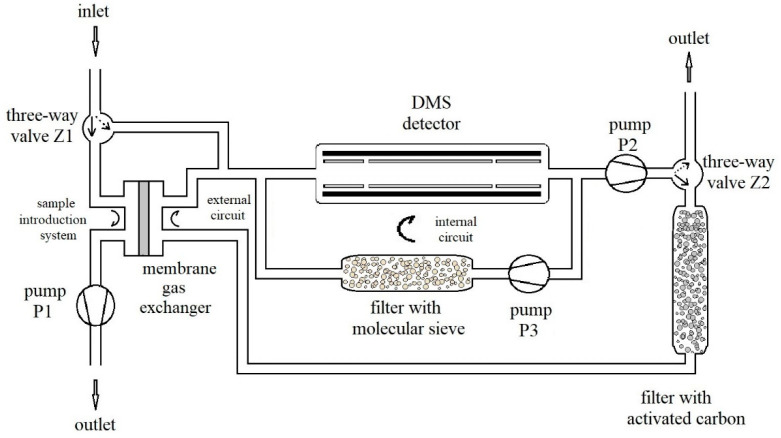
Diagram of the measurement system.

**Figure 5:**
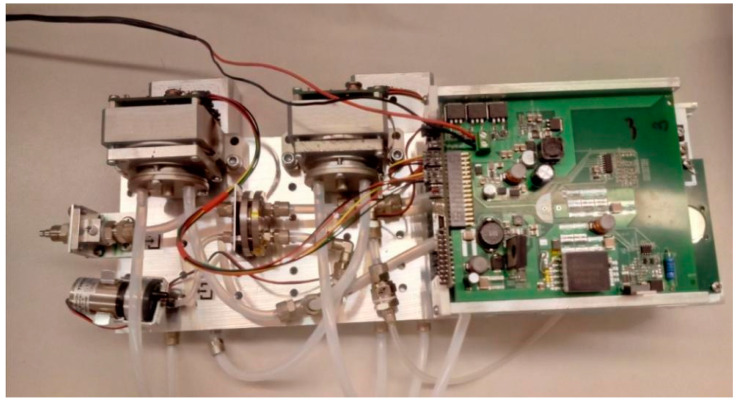
The measurement system.

The gas system is presented in Figure 4. The measurement system was constructed at WIChiR (Warszawa) for the purpose of detection and identification of TATP and HMTD (Figure 5). The system was constructed with the use of three rotation pumps (Thomas) (P1, P2, P3) with flow rates respectively of 1, 1, 3.2 L/min and two three-way valves (Z1, Z2). The advantage of the constructed system is the possibility to work in the membrane mode when detecting substances with high volatility and in the membrane-free mode when detecting substances with low volatility, such as HMTD.

In the membrane mode, the Z1 valve is switched to gas flow through the membrane, which is forced by the P1 pump at this time. The Z2 valve at the P2 pump outlet allows gas to flow through the carbon filter, and then the sample is taken from the membrane exchanger (sample introduction system), and goes to the DMS.

In the membrane-free mode, the gas to be analyzed is charged directly to the DMS. Setting the valve Z1 to take air allows direct sampling from the test area.

The P2 pump allows you to regulate the flow with the required amount of gas for analysis (external circuit). In this mode, the valve Z2 at this time is switched over to allow gas to be purged after the analysis.

The P3 pump is used to regulate the carrier gas flow in the DMS chamber at 3.2 L/min (internal circuit). The applied molecular sieves clean the gas in the internal circuit of the DMS chamber.

Due to the high vapor pressure of TATP, most measurements for this substance are made in the membrane mode, while for HMTD all measurements are made in the membrane-free mode. In the membrane mode, the appearance of the HMTD peaks was not recorded as the intensity of peaks was below the limit of detection (LOD). TATP, HMTD, and DADP were obtained synthesized at the Military Institute of Armament Technology (Zielonka).

## 3. Results

The gas system presented in Figure 4 was used to analyze pure TATP, DADP, HMTD and a mixture of TATP and DADP. Scattering spectra were recorded for a separation voltage of 118 Td.

**Figure 6:**
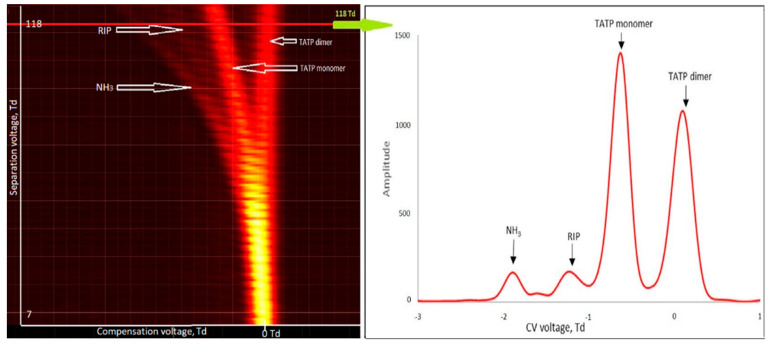
Dispersion plots for positive ions of triacetone triperoxide (TATP) (500 µg/m^3^) and drift spectra for 118 Td.

For TATP (99% purity), monomer and dimer ions were obtained in positive mode (Figure 6). The monomer ion peak was issued at −0.6 Td and the dimer ion peak at +0.1 Td. No peaks for negative ions were observed.

**Figure 7:**
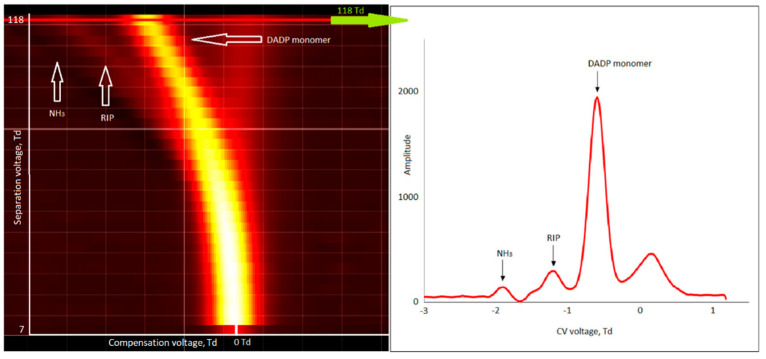
Dispersion plots for positive ions of diacetone diperoxide (DADP) (600 µg/m^3^) and drift spectra for 118 Td.

For TATP (99% purity), monomer and dimer ions were obtained in positive mode (Figure 7).

Traces of dimer ions were observed for DADP (low intensity compared to monomer ions). When increasing the DADP concentration, the RIP and NH_3_ peaks disappeared. This means that the peak intensity of the DADP monomer increased but the peak of the DADP dimer did not increase.

**Figure 8:**
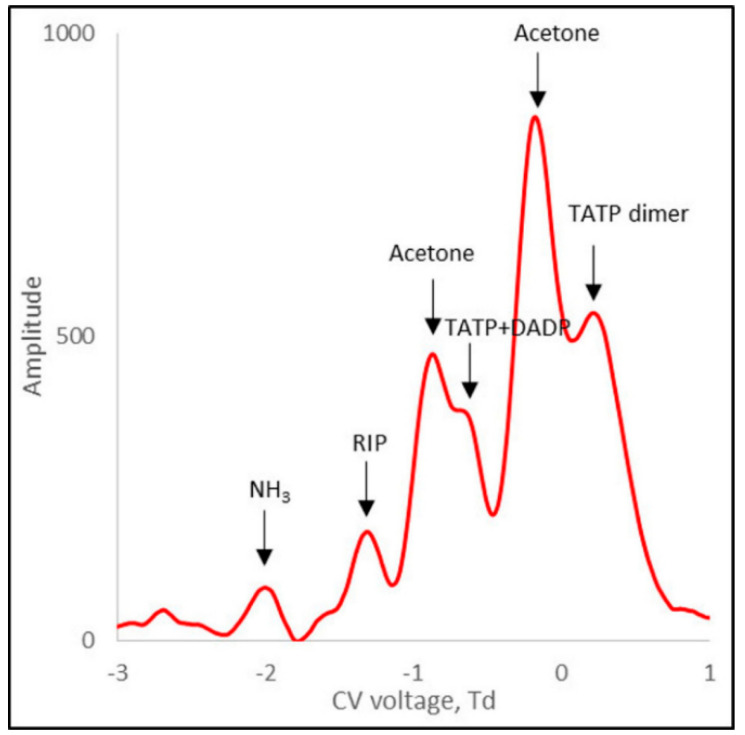
Differential ion mobility spectrometry (DMS) spectrum for 118 Td for TATP positive ions, with NH_3_ at a concentration of 50 ppb in the internal circuit.

**Figure 9:**
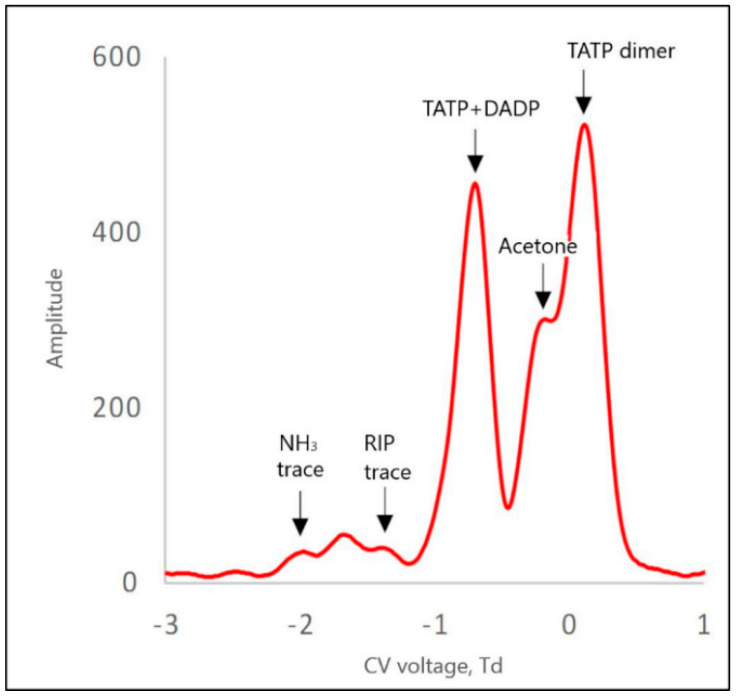
DMS spectrum for TATP positive ions, with NH_3_ at a concentration of 200 ppb in the internal circuit.

In further studies, a mixture of TATP and DADP (approximately 78% *w*/*w* TATP) was analyzed. This research doped NH_3_ at two concentrations (50 ppb and 200 ppb) in the internal circuit. The results for 118 Td are shown in Figure 8 and Figure 9.

In the case of a TATP + DADP mixture for positive ions, there were generally four peaks: one from the TATP + DADP monomer, the other from the acetone monomer, and two peaks from the dimer ion, from acetone and TATP. Due to the rather low resolution of DMS, the peaks derived from the TATP and DADP monomers were not separated and appeared as one peak. In the presence of a low concentration of ammonium, the dominant peaks were those of acetone (Figure 9).

For a system with a 200 ppb ammonium concentration, two reaction peaks were visible: one for ammonium and the other for H^+^(H_2_O)_n_, both of which showed small amplitudes. The ammonium peak was only twice as high as the noise.

**Figure 10:**
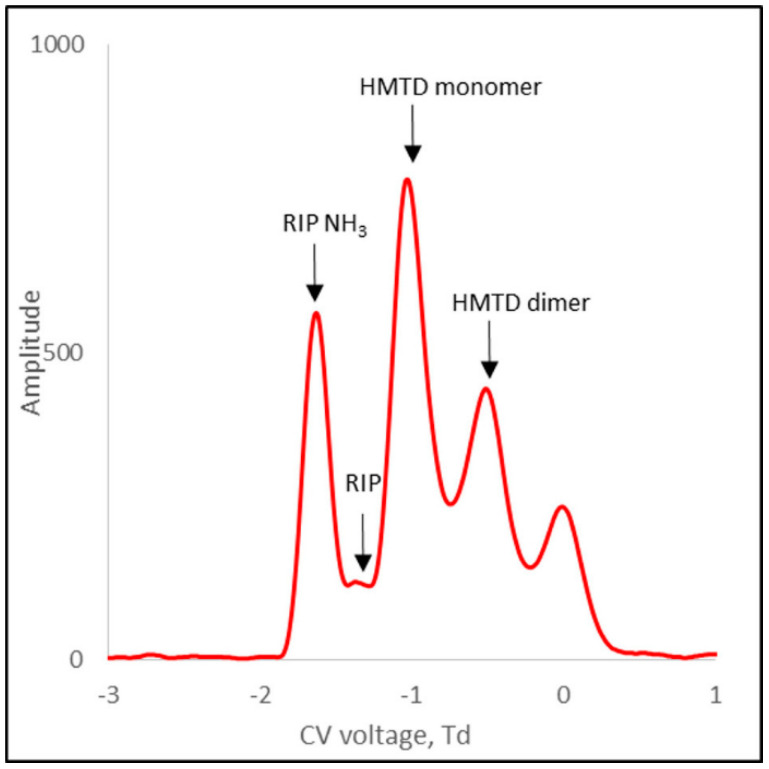
DMS spectra for HMTD positive ions.

From the spectra presented in Figure 9, we can see that acetone significantly disturbed the accuracy of the measurement. In particular, the acetone dimer peak interfered with the analysis. The acetone peaks were higher than the TATP peaks. Doping the carrier gas with 200 ppb NH_3_ caused a decrease in the intensity of the acetone peak (Figure 10). Therefore, it can be concluded that the minimum value of ammonia concentration in the carrier gas, which is necessary for the separation of the peaks from TATP, was approximately 200 ppb.

Figure 10 shows the spectrum for HMTD positive ions. For this substance, both monomer and dimer ions were formed. Table 1 presents the compensating voltage for all positive ions.

To determine the possibility of detecting the mixture of TATP and HMTD, tests were carried out with the DT-IMS detector. The tests were carried out under the same conditions as with a DMS detector, with concentrations of TATP totaling 250 µg/m^3^ and 5 ppm NH_3_ in the internal cycle.

**Figure 11:**
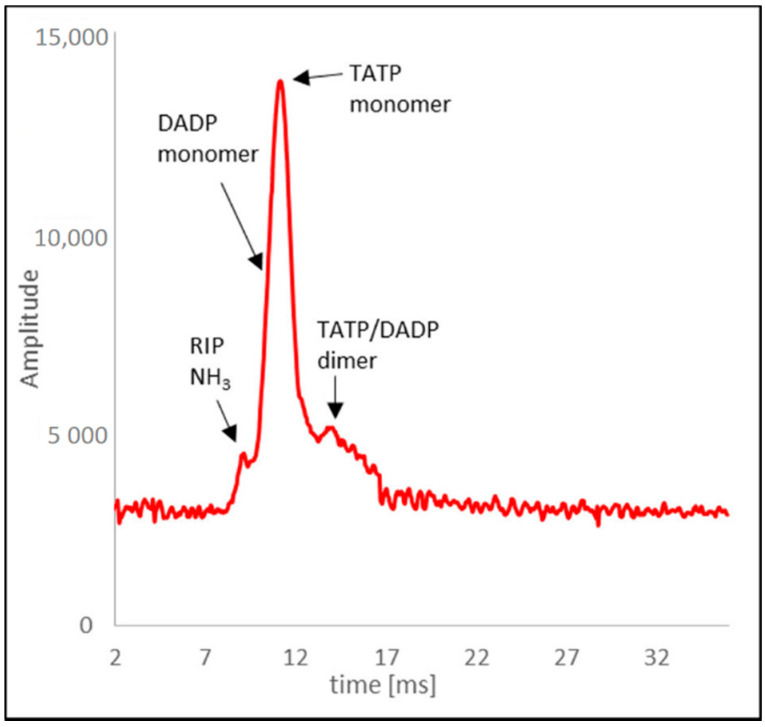
Drift time spectrum of TATP.

The spectrum showed (Figure 11) the RIP peak with NH_3_ and the most insensitive TATP monomer peak. The DADP peak was invisible (it appeared on the leading edge of the TATP monomer peak). One dimer peak was observed as a mixture of TATP and DADP ions. In the case of recording the spectrum for DADP, peak separation was not observed, and only the shift of the peak from RIP + DADP ions towards increasing time values from 9.3 ms to a maximum of 9.7 ms was observed. Table 2 shows drift times for the mixture of TATP and DADP for a concentration of 250 µg/m^3^.

In the measurement system shown in Figure 2, no recorded peaks from HMTD were observed. After removing the inner membrane, the spectrum showed a peak with reduced mobility of approximately 1.5 cm^2^/Vs. HMTD detection using DT-IMS was possible only under laboratory conditions (for dried air).

## 4. Discussion

The acetone residues reduced the detection capability of the DMS detector. The PA for acetone is 812 kJ/mol [38], which is lower than the PA of the added ammonia (853.6 kJ/mol [39]); therefore, the use of ammonium as an admixture in the carrier gas will help to reduce the appearance of acetone peaks. When detecting improvised explosives using differential ion mobility spectrometry, the carrier gas should be doped with a minimum of 200 ppb NH_3_ to reduce the effect of acetone (TATP contamination). During the analysis of TATP containing DADP as an impurity, an increase in the amplitude of the monomer was observed, the peak of which was at the same location as that of the TATP monomer, without a significant increase in the dimer amplitude. This may indicate a lack of separation of the dimer for DADP.

In the case of TATP measurements with the DT-IMS detector, the measurement system used a carrier gas with NH_3_ content at the level of several ppm. The appearance of a peak close to the reaction ions (NH_3_) was observed in both the TATP and DADP studies.

Differential ion mobility spectrometry was more effective than DT-IMS. For DT-IMS, the peaks derived from TATP and DADP were located close to the peaks of the reaction ions; in the case of DMS, the peaks were spaced apart and it was possible to separate them more efficiently. Moreover, for the gas system with the membrane, no HMTD peaks were observed in the case of IMS. Furthermore, it is impossible to detect HMTD in generally used DT-IMS systems with membranes. In this case, there was no peak observed from the analyte. 

In the DMS studies, it was observed that the peaks moved towards higher compensatory voltages in membrane-free mode, due to a humidity effect. When adjusting the effect of humidity on the location of peaks, the identification of HMTD and TATP was maintained at the same level—no shift of adjusted peaks was observed.

The response time for instruments with membranes for TATP is a few seconds, while that for HMTD is from several dozen seconds to two minutes. The response time for HMTD results is the time required for membrane permeation, whereas for a membrane-free system, TATP and HMTD are detected in seconds.

## 5. Conclusions

Detection of triacetone trioxide and hexamethylene triperoxide diamine can provide early warning of a terrorist threat. DMS is currently one of the leading new technologies for the separation and detection of chemicals in the gas phase. 

When we used the membrane-free mode, the differential ion mobility spectrometry detected TATP and HMTD correctly. 

The use of ammonia as the dopant carrier gas in system with and without membrane allows for the detection of inaccurately purified TATP containing the by-product DADP and residual acetone. The use of ammonia in the carrier gas as a dopant helps in TATP analysis for a membrane or membrane-free system.

Due to the differences resulting from the vapor pressure, it is necessary to use both a membrane and membrane-free system at the same time. In the case of the membrane-free mode, it is necessary to apply a peak position correction due to the variable value of wetness.

Regarding laboratory analysis, the detection times of TATP and HMTD are short and allow for quick scanning tests. Such a system allows the detection of HMTD at a level lower than that of the gas chromatography used in WIChiR. 

This study is the first to report detection of TATP and HMTD by differential ion mobility spectrometry.

## Figures and Tables

**Table 1:** Compensating voltage values for positive ions.

Substance	Positive Ion	CV (Td)
TATP 99% purity	RIP	−1.3
	monomer	−0.6
	Dimer	0.1
DADP 99% purity	RIP	−1.3
	monomer	−0.6
	dimer	0.1
TATP + DADP (50 ppb NH_3_)	RIP	−1.3
	acetone monomer	−0.9
	acetone dimer	−0.2
	TATP + DADP	−0.7
	TATP dimer	0.2
TATP + DADP (200 ppb NH_3_)	RIP	−1.3
	acetone monomer	−1.0
	acetone dimer	−0.2
	TATP + DADP monomer	−0.6
	TATP dimer	0.1
HMTD	RIP	−1.3
	HMTD monomer	−1.0
	HMTD dimer	−0.5

**Table 2:** Drift time for TATP and DADP ions.

Positive Ion	Time (ms)
RIP (NH_3_)	9.3
DADP monomer ion	9.7
TATP monomer ion	11.2
TATP + DADP dimer ion	13.2

## Data Availability

Data sharing not applicable.
